# The effect of controlling person’s illegalities on stock price returns: Evidence from Elman neural network model

**DOI:** 10.1371/journal.pone.0266741

**Published:** 2022-04-20

**Authors:** Ming Xiao, Ying Guo, Xionghui Yang, Ge Li, Moustafa Mohamed Nazief Haggag Kotb Kholaif

**Affiliations:** 1 School of Economics and Management, University of Science and Technology Beijing, Beijing, People’s Republic of China; 2 CITIC Group Corporation, Beijing, People’s Republic of China; University of Almeria, SPAIN

## Abstract

Controlling persons are the ultimate decision-makers of listed companies. Their illegalities have impacts on investors’ wealth, firm development, and capital market’s quality. Against this backdrop, we provide a quantitative analysis of the short-term stock price reaction to the criminal detention announcements of controlling persons throughout 2007–2019. We applied the Elman neural network (ENN) model into the classical event study methodology and demonstrated that the combination of them helps to improve the estimation accuracy of the stock price reaction. The results show that the stock price has a significant negative reaction to the criminal detention announcements of listed companies’ controlling persons on the announcement day, and the average reaction level is -6.67%. Additionally, the crisis communication measures of the firms could diminish the negative impact of such mandatory disclosure information on their stock price, but the effect is limited. Finally, the 31 companies in our sample cause a total loss of RMB 21.1 billion in market capitalization on the announcement day alone. The above results indicate that the impact of listed companies’ controlling persons on the capital market is tremendous, although the number of this group is small. Our work enriches the listed companies’ illegalities research and provides a reference for investors’ investment choices and follow-up decision making of regulatory authorities. It also provides some guidance for most of the researchers to further explore the application of data mining techniques in nonlinear problems.

## Introduction

Emerging capital markets have developed rapidly and become rising stars in the world economy in recent years. As the largest emerging country, China has made considerable development in its capital market in the past three decades, playing a crucial role on the world economic stage and becoming the focus of the world’s attention. However, the issues of irregular operation, governance of listed companies, and poor development quality of the capital market are still prominent in China (Opinions No. 14(2020) on Further Improving the Quality of Listed Companies). In 2019, 14 controlling persons of listed companies were criminal detained, making it the largest “black swan flock” in the Chinese capital market. The frequent occurrence of “black swan events” has received widespread attention from all sectors of society [1]. As a result, the ensuing stock market shocks have seriously affected investors’ confidence and posed a great threat to the smooth operation and sustainability of the capital market.

In mature capital markets, market capitalization management is equivalent to value management. When the controlling person becomes a “black swan” in a listed company, it can be a fatal disaster for its value management [2]. Therefore, identifying the market value loss caused by the illegality of controlling persons is of great realistic warning and reference significance. Against this backdrop, we wonder when and how the stock market reacts to controlling persons’ illegalities of listed companies. The previous works of controlling persons’ illegalities mainly use news or analytical reports for analysis [1, 3], and all of the studies about the market reaction of listed company’s illegalities are from firm’s or senior executive’s aspect [4–8], none of them concerning the market reaction to controlling persons’ illegalities.

To shed light on the market reaction to controlling persons’ illegalities of listed companies, we perform a quantitative analysis through detailed multi-case analysis using stock price data and the Elman neural network (ENN) model combined with the event study method. Specifically, we investigate the short-term stock price reaction to criminal detention announcements of listed companies’ controlling persons, which further provide targeted recommendations and empirical evidence for supervisors to make subsequent regulatory decisions. Our sample includes 31 companies listed in the Chinese stock market whose controlling persons were criminal detained during the 2007–2019 period.

First, the disclosure of bad news will lead investors to reevaluate their judgment on the firm’s internal control quality and management level and thus revise their perception of the firm’s risk level and expectation about its future profitability [9]. Based on the potentially devastating nature of crisis events, we expect to observe a negative stock price reaction to criminal detention announcements of controlling persons. Consistent with our expectations, the results show that the abnormal return (AR) on the announcement day is statistically significantly negative. More specifically, the mean AR measured by the ENN model is -6.67% (two-tailed P = 0.0001). We also perform a robust test using a traditional market model and the reaction is still negative. This suggests that the stock market has a significant negative reaction to the criminal detention announcements of listed companies’ controlling persons on the event day.

Second, with the general improvement of listed companies’ crisis management, the involved firms will carefully consider the disclosure timing and method to diminish the negative effect. Thus, they may promptly adopt crisis communication measures in the face of such mandatory disclosure information. To control the confounding effect of such measures, we only consider the sample of 19 firms without other material information and examine the market reaction again as a robustness test. We find that the average AR on the announcement day is still significantly negative, but the absolute value increases 1.37%, which is -8.04%. This reaction level is also economically significant, compared with the negative level caused by corporate illegalities in other countries found in the existing literature [4, 5, 8, 10]. This suggests that the confounding announcements issued by listed companies can diminish the negative impact on their stock prices.

Third, we conduct a statistical analysis on the market capitalization loss caused by the criminal detention announcements of controlling persons. The results reveal that the 31 firms caused a total loss of RMB 21.1 billion to the Chinese capital market on the announcement day alone, which suggests the impact of controlling persons of listed companies on the capital market is tremendous, although the group size of them is relatively small.

Our paper contributes to the literature in three ways. First, even though several studies have examined the market reaction to listed companies’ illegalities [4–8], none of them concern the controlling person’s impact. We fill the gap by shedding light on the illegalities of controlling person. Second, we are the first to quantitatively analyze the market reaction to controlling persons’ illegalities in the context of Chinese listed companies. Because China is the largest emerging market and the second-largest economy globally, our work may guide decision-making towards the subsequent regulations and have important implications for other countries. Third, we combine the ENN model with the event study method to measure the market reaction, which overcomes the linearity assumption bias of the classical normal return (NR) estimation models and improves the reliability of findings. Our research provides guidance for most researchers to further explore the application of data mining techniques in nonlinear problems.

The remainder of this paper is organized as follows. Section 2 is the related work review. Section 3 presents the data and methodology. Section 4 reports the empirical results, and Section 5 concludes.

## Related work

### Definition of controlling person

The controlling person of a listed company is a person, including a natural person, legal person, or other organizations, who can actually dominate the company’s behavior through an investment relationship, agreement, or other arrangements. The controlling person’s control over a listed company involves three levels: the shareholders’ meeting, the board of directors, and the managers. In practice, the controlling person often achieves the control through equity advantages, strategic decisions, and personnel arrangements for key positions and greatly impacts the company’s internal governance and financial decisions [11].

Based on the perspectives of equity concentration and ultimate control of Chinese listed companies, the controlling person is universal in listed companies [12]. However, in the early stage of joint-stock enterprise development, only the controlling shareholder of a listed company was required to disclose information related to them, which leads to a growing number of scandals in which the controlling person behind the scenes uses the organizational form of company to commit securities fraud and evade liability. To prevent such illegal events, the laws use the concept of “controlling person” and increasingly focus on disclosing the ultimate beneficial owner behind the listed company. According to relevant regulations of China Securities Regulatory Commission, the listed companies should disclose the information in the form of interim announcements if it is suspected of violating the law and is investigated by the competent authorities or is subject to criminal punishment or major administrative punishment.

### Market reaction to listed companies’ illegalities

The grave corporate illegalities (e.g., the financial fraud event of Enron in the United States in 2001 directly led to the promulgation of the Sarbanes-Oxley Act.) have an enormous negative influence on the smooth operation of stock markets and the wealth of investors, resulting in growing studies on the market reaction to such events. From the perspective of the subjects of illegalities, these illegal events roughly include corporate illegalities and individual illegalities of senior executives. The market reaction level of individual illegalities of senior executives is between -0.95% and -1.80% found in existing literatures. However, the controlling person does not belong to the senior executives of a listed company, and to our knowledge, there are no researches about the market reaction to their illegalities at present. In the following, we briefly review the main studies according to the above classification to show this research gap and the shortcomings of the traditional estimation methods on market reaction.

#### Corporate illegalities

In the previous literature about corporate illegalities, most of the studies mainly focus on the market reaction to the enforcement actions of securities regulators, such as the U.S. Securities and Exchange Commission and China Securities Regulatory Commission [4, 5, 13–15]. However, these studies distinguish neither the enforcement stages nor the specific illegality types.

Recently, some researchers made divisions of the enforcement procedures and examined the market reaction to the enforcement announcements issued at different stages [10–14, 16–20]. They find that the more advanced the regulatory procedure, the higher the negative market reaction level triggered by the enforcement announcements. For instance, Feroz et al. examine the market reaction to a total of 224 Accounting and Auditing Enforcement Releases issued by the U.S. Securities and Exchange Commission between April 1982 and April 1989 [14]. Among them, the cumulative abnormal return of 58 Accounting and Auditing Enforcement Releases in the [–1,0] window around the investigation announcements is up to -7.50%, while the stock prices around the window of investigation result announcements do not change significantly. Karpoff et al. find that the average AR on the event trigger day when the company voluntarily discloses its potential problems is -25.24%, on the regulatory investigation announcement day is -14.41%, and on the punishment announcement day is -6.56% [10]. Researches based on Chinese data also discovered similar conclusions. The market reaction to the punishment announcements is about -2.00% [13, 17–19], and the market reaction to the regulatory investigation announcements is much higher than it, which is about -6.00%~-8.00% [19, 20]. Nonetheless, some scholars reach the exact opposite conclusions. For example, Wu and Gao find a significant positive stock price reaction to the punishment announcements on the event window after the announcement day [16].

Furthermore, Davidson investigates the different types of listed companies’ illegalities [21]. His results show that the overall market reaction is insignificant, while the stock price reacts remarkably to the announcements of bribery, tax evasion, and violations of government contracts. Blaufus et al. examine the market reaction to 176 news about corporate tax strategies [8]. By distinguishing the news into tax avoidance(legal) and tax evasion(illegal), they find a negative stock price reaction in the event window around the disclosure of tax evasion news, while the conclusion for the former is not clear. What is more, some scholars who study the market reaction to listed companies’ environmental illegalities observe a significant negative reaction [22–24]. By contrast, the reaction level of the Chinese stock market to such similar events is lower than that of other countries [25].

#### Individual illegalities of senior executives

Regarding the economic consequences of senior executives’ illegalities, the existing literature mainly focuses on corporate performance [26, 27], investment efficiency [28–30], bankruptcy liquidation [31, 32]. The few studies on the market reaction to senior executives’ illegalities all find a negative reaction. For example, Zhang and Guan classify the types of individual illegalities of senior executives and investigate the market reaction to 283 cases of senior executives’ embezzlement crimes from 1991. They find that senior executives’ embezzlement crimes lead to a significantly negative market reaction in the short-term, and the institutional investors can also significantly influence this reaction level [33]. Quan and Yu [7], Jiang and Zhao [6] also explore the market reaction to announced senior executives’ illegalities, whose samples are 307 cases from 1997 to 2010 and 170 announcements from 2000 to 2015, respectively. Both find that disclosing such illegal information causes a significant decline in listed companies’ stock prices.

To sum up, it is necessary to investigate the market reaction to controlling persons’ illegalities. As the person who has “control” over a listed company, the controlling person is like the “brain” of that firm and has a profound influence on its business decisions. Based on the previous analysis, it can be believed that, on average, the stock price reaction to the criminal detention announcements of controlling persons is negative. However, what is the level of the negative reaction? When does the reaction start? How much does the criminal detention announcements knock off investors’ wealth? Research on these issues is still in its infancy. This paper aims to provide answers to the above questions through quantitative analysis.

### Market reaction measurement methods

This paper uses the event study method to measure the market reaction brought by the illegalities of the controlling person. The event study method refers to using financial market data to determine the impact of a specific economic event on the value of a listed company. The event can be a general economic event (e.g., inflation, a trade deficit, enactment of a policy decree) or a major company event (e.g., merger and acquisition announcement, bond issue, dividend declaration). Due to its clear research logic and general applicability in various types of events, the event study method has been widely used by scholars globally and is suitable for examining the market reaction to the controlling person’s criminal detention announcement. Abnormal return (AR) and cumulative abnormal return (CAR) in the event window are indicators of the market reaction used in the event study method. Where AR refers to the difference between the normal return (NR) and the realized return in the event window, NR refers to the counterfactual expected return (*E(R*_*it*_*)*) of the company’s stock price if the event does not occur. Consequently, the key step of AR calculation is to estimate NR accurately, and the improvements scholars made to the event study method are mainly around NR estimation since it was proposed.

However, the dominant NR estimation models which are based on linear assumption and regression analysis are biased, due to the nonlinearity of the stock price series and the relationship between individual stock returns and market returns [34, 35]. It’s difficult to express the nonlinear relationship with a definite functional analytic, but this is where artificial neural network (ANN) excels. In particular, the applicability and effectiveness of the ENN in time series forecasting have been widely proven, and it is the motivation of combining the ENN with the event study in this study.

#### Traditional NR estimation methods

There are many methods for NR estimation, which can be commonly classified into the following four categories: unadjusted return, mean-adjusted return, risk-controlled portfolio return, and risk-adjusted return. First, the unadjusted return directly uses the realized return as AR, thus assuming NR is 0. The mean-adjusted procedure defines NR as a constant *k*. These two methods are easy to calculate, but both of them ignore the differences among individual stock returns. The third category is the risk-controlled portfolio method used by Gonedes et al [36]. Based on the pre-estimated stock price reactions, this method groups firms and assigns weights to them, and calculates the weighted average return of all firms in the portfolio. The difference between this return and the market return is the AR of the portfolio. The last category of estimation methods covers a wide range of risk-adjusted models, such as the market model, market-adjusted model, multi-factor model, etc, which are developed based on the capital asset pricing model. Brown and Warner prove that the estimation results of the market model, market-adjusted model, and mean-adjusted model have very little difference, and they are all superior to the risk-controlled portfolio model [37]. In addition to the market factor, scholars try to find other factors that affect stock return to improve the explanatory ability and forecast accuracy of the risk-adjusted models, and put forward the multi-factor models, but with little success. The explanatory ability of the newly added influencing factors is generally weak, and the improved models still do not overcome the limitation of the linear relation assumption. However, in the test of the applicability of the capital asset pricing model in the Chinese stock market, Jin and Liu prove that the relationship between individual stock returns and market returns is nonlinear [35]. Hence the traditional linear models have specification bias.

Table 1 summarizes some important researches about the market reaction of listed companies’ illegalities. It can be seen that in relevant studies at domestic and abroad, the most widely used NR estimation model is the market model, and the stock price reactions found are mainly negative. For the Chinese listed companies’ illegalities research, the measured negative market reaction ranges from -0.95% to -8.00%.

**Table 1:** Summary of prior studies on the market reaction to listed companies’ illegalities.

Author(s)	Year	Sample Period	NR Estimation Model	Market Reaction
[4]	1983	1970–1980	Portfolio model	-2.82%
[5]	1988	1970–1988	Market model	-0.87%
[14]	1991	1982–1989	Market model	-7.50%
[21]	1994	1965–1990	Market model	-5.13% ~ -0.69%
[22]	1995	1989	Market model	-0.28%
[16]	2002	1999–2000	Market model	0.50%
[13]	2005	1999–2003	Risk adjustment model	-1.87% ~ -1.12%
[10]	2008	1978–2002	Portfolio model	-25.24% ~ -6.56%
[18]	2011	2008–2010	Control company	-2.70%
[19]	2014	2002–2011	Market model	-2.00% ~ -6.00%
[23]	2017	2000–2015	--	-2.04%
[24]	2018	2015–2016	Market model	-0.69%
[8]	2019	2013–2016	Market model	insignificant
[20]	2019	2007–2015	Market model	-8.00%
[33]	2017	1991–2017	Market model	-1.10%
[7]	2017	1997–2010	--	-0.95%
[6]	2017	2000–2015	Market model	-1.80%

#### The artificial neural network method

With the continuous improvement of chaos and fractal theory, using data mining techniques to solve complex nonlinear problems in the financial field is attractive. ANN is an information processing method established by imitating the human brain’s neural network structure and function. It consists of a large number of interconnected processing units, i.e., neurons. The ANN method does not need to preset the mapping relationship between input and output data. It uses the initial connection weights randomly assigned among neurons to fit the relationship behind the input data. After gradually adjusting the connection weights to minimize the sum of squared errors through repeated learning and training, information processing can be achieved, and the actual relationship between input data and output data is fitted. ANN can approximate any nonlinear function with arbitrary precision [38, 39], becoming an effective way in financial time series forecasting field [40–42]. Many studies demonstrate that combining ANN with other statistical or machine learning techniques can improve the forecast accuracy compared with the traditional prediction methods [43–45].

**Fig 1:**
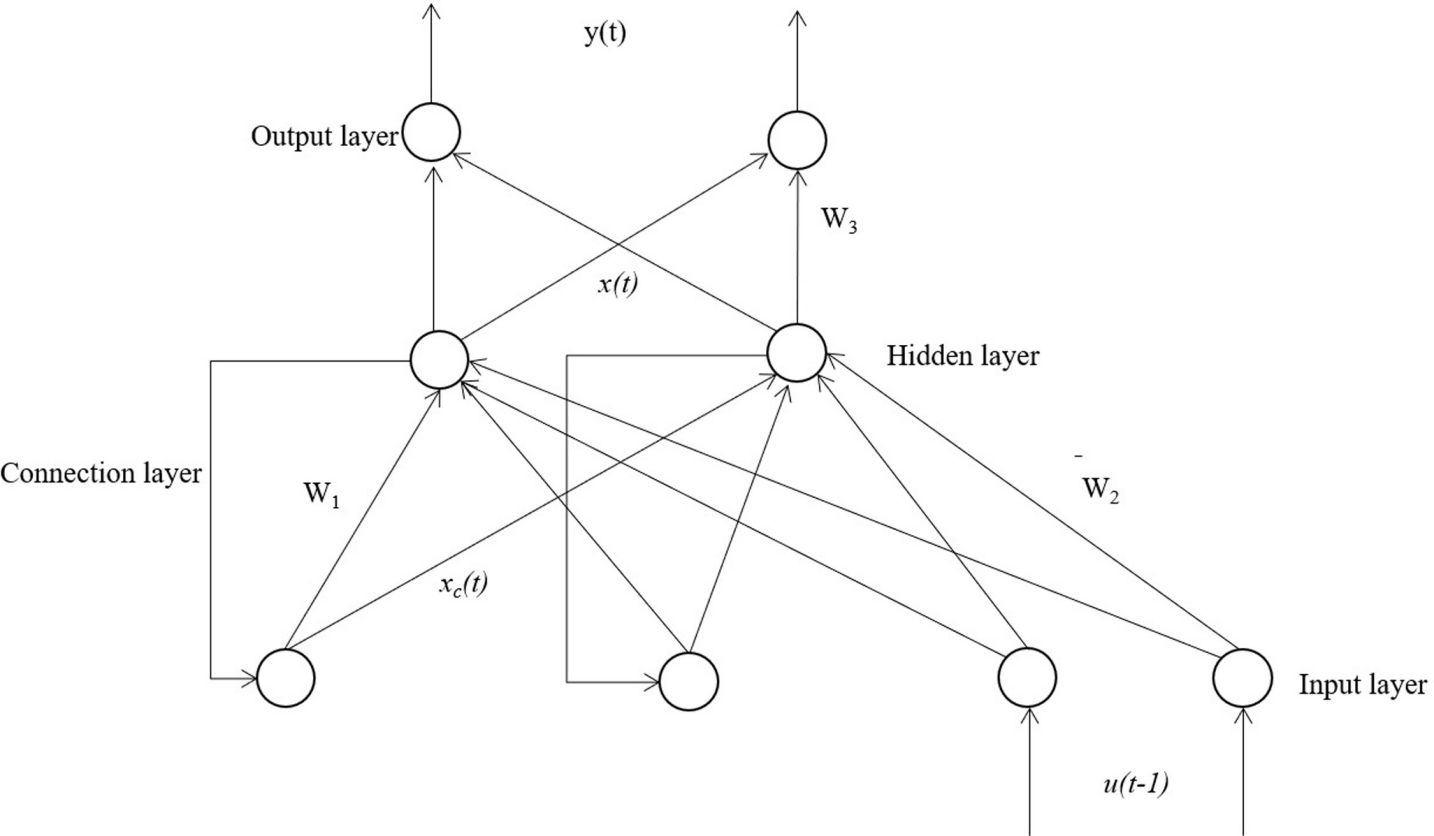
Structure diagram of Elman neural network.

**Fig 2:**
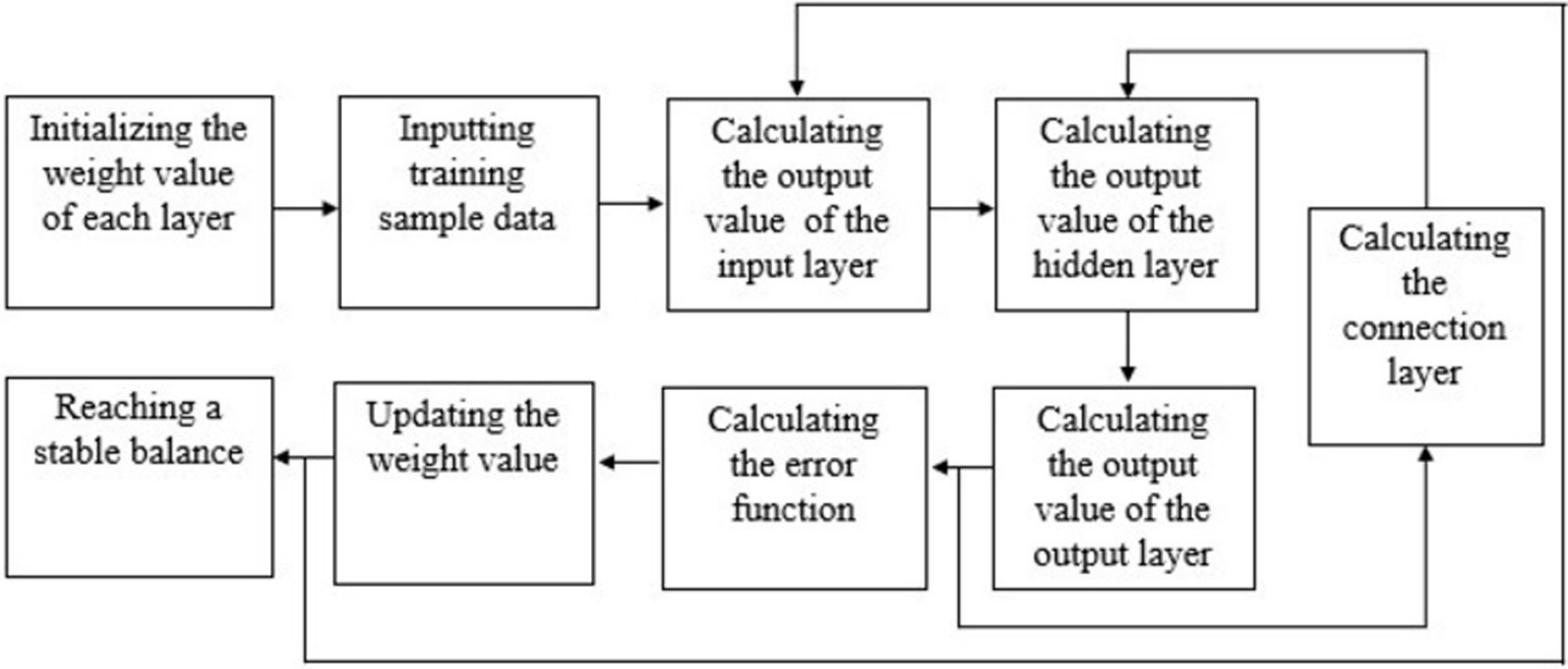
Operation logic of Elman neural network.

In particular, the Elman recurrent neural network (ENN) has shown great promise in many different types of time-series analysis. ENN is a dynamic recurrent neural network first proposed by Elman in 1990 for speech processing problems [46]. It consists of the input, hidden, connection, and output layer (Fig 1). The input information enters the hidden layer neurons through the input layer neurons. The output information of the hidden layer is calculated and stored by the connected layer neurons, and then enters the hidden layer as input information again. This process repeats iteratively until the error function and the weight reach a stable balance state. Finally, the output layer weights the data and outputs it (Fig 2). ENN uses the newly added connection layer as the delay unit of the calculation process to self-link the calculation results to the calculation process for relearning, so that it has the function of dynamic memory and high-speed optimization solution.

Jalal et al. [47] used the ENN and the NARX Neural Network for forecasting call volumes in call centers. Also, Kolanowski et al. [48] presented a navigation system based on an ENN and data fusion from different sensors. Zheng [49] applied an ENN in forecasting the opening prices of the Shanghai Stock Exchange. Wu and Duan used the ENN to predict stock [50] and gold future markets [51]. Xiao et al. take the ENN to measure the short-term merger performance of Chinese listed companies [52]. All the results of the above studies indicate that the ENN model is more efficient in time series forecasting. Furthermore, Wang et al. introduce direct input-to-output connections (DIOCs) into the ENN for stock index forecasting [34]. DIOCs refer to adding a linear component in the input-to-output mapping. It helps to improve forecasting accuracy when there are both linear and nonlinear components in the researched data [53–55]. However, Wang et al. also point out that in cases where the problem at hand is entirely nonlinear, DIOCs should not be used [34].

This paper shall use a standard ENN model to measure the market reaction to controlling persons’ criminal detention announcements. To improve the robustness of our findings, the most prevailing market model of the traditional models to estimate the market reaction is also used.

## Data and methodology

### Sample selection

To form our sample, we began with the Chinese listed companies whose controlling persons were criminally detained during the 2007–2019 period. We first searched all of the listed companies’ announcements using the keywords “arrestment” and “detention”. And then, we searched and identified the first disclosure of each announcement, and only this first disclosure was included in our sample. Based on these two procedures, 41 cases were obtained as the initial research sample. After that, we excluded 2 cases in which the company’s stock was suspended due to other major matters on the announcement date because the stock price data is needed to measure the market reaction. In addition, given the peculiarities of the financial behavior of loss-making companies, we excluded 8 cases that happened in ST firms. Finally, the research sample of this paper contains 31 valid observations. The stock codes of the sample firms are as below: 600634,600678, 000639, 002200, 000782, 600381, 002161, 000510, 300101, 002081, 300209, 002628, 002629, 300202, 002676, 002447, 002680, 300028, 300176, 002737, 002290, 601519, 002517, 601155, 600083, 300431, 300187, 002098, 002547, 603466, 600738. Table 2 shows the process of collecting the criminal detention announcements of listed companies’ controlling persons.

**Table 2:** Overview of the sample selection process.

Selection procedures	Observations
Criminal detention announcements of listed companies about the controlling person	41
*Less*:	
Observations for firms being in the suspension period because of other major matters	2
Observations for firms marked as ST or *ST by CSRC	8
Final sample	31

Note: ST system, i.e., special treatment, is a unique risk warning system in the Chinese stock market. Announced on April 22, 1998, the system refers to prefixing the word of ST or *ST to the listed company’s abbreviation with abnormal financial and other statuses as a risk warning to investors. The abnormal status mainly refers to the following two situations. The first one is the audited net profit of the listed company in the two fiscal years is negative, and the other one is that the audited earning per share of the listed company in the most recent fiscal year is lower than the book value of the stock. In the Chinese stock market, the daily trading limit for ST firms is ±5%, while the limit for non-ST firms is ±10%.

Table 3 reports the illegal types (Panel A), the annual distribution (Panel B), and whether there were other announcements except for criminal detention announcement, on the event day (Panel C) of our sample. Undermining the finance order, bribery, and illegal operation are the top 3 illegal types. They account for 70.59% of the total sample. What’s more, the number of criminal detention events of controlling person increased sharply in 2018 and 2019, accounting for 54.84% (17/31) of the total sample. Moreover, on the event day, 12 firms did not issue any other announcements, 7 firms issued other announcements without confounding events, and 12 firms issued other announcements with confounding events.

**Table 3:** Sample distribution.

Categories	Number of Observations	Percentage
**Panel A: Illegal types**		
Undermining the finance order	9	26.47%
Bribery	9	26.47%
Illegal operation	6	17.65%
Embezzlement crimes	2	5.88%
Contract/finance swindling	2	5.88%
Crime of breach trust	1	2.94%
Others	5	14.71%
Total	34	100.00%
**Panel B: Annual distribution**		
2007	1	3.23%
2008	0	0.00%
2009	2	6.45%
2010	0	0.00%
2011	2	6.45%
2012	2	6.45%
2013	2	6.45%
2014	2	6.45%
2015	2	6.45%
2016	0	0.00%
2017	1	3.23%
2018	4	12.90%
2019	13	41.94%
Total	31	100.00%
**Panel C: Announcements on the event day**		
No other announcements	12	38.71%
Other announcements without confounding events	7	22.58%
Other announcements with confounding events	12	38.71%
Total	31	100.00%

Note: Undermining the finance order includes market manipulation, insider trading, fraudulent issuance, and illegal information disclosure. Some controlling persons were charged with more than one count, so the total number of illegalities is higher than the number of announcements.

The object of our study objectively dictates a relatively small sample size for this paper, as the group of controlling persons is not as large as the group of senior executives. The common concern regarding small samples is the lack of statistical power. However, Kothari and Warner suggest that if ARs concentrate in a short period, event studies using very small samples can yield significant results [56]. Furthermore, Cready and Hurtt note that many short-window event studies used small samples [57]. They examine event studies using short window metrics, published in The Accounting Review during 1992–2001. Of the 35 studies examined, 16 (46%) contain at least one analysis using fewer than 50 observations. Finally, statistical power is not an issue for this study because we find statistical significance.

The announcement data required for this study are collected manually from the websites of the Shanghai Stock Exchange (http://www.sse.com.cn/disclosure/listedinfo/announcement/) and Shenzhen Stock Exchange (http://www.szse.cn/disclosure/listed/notice/index.html). The stock price data are obtained from the Wind database.

### NR estimation methods

**Fig 3:**
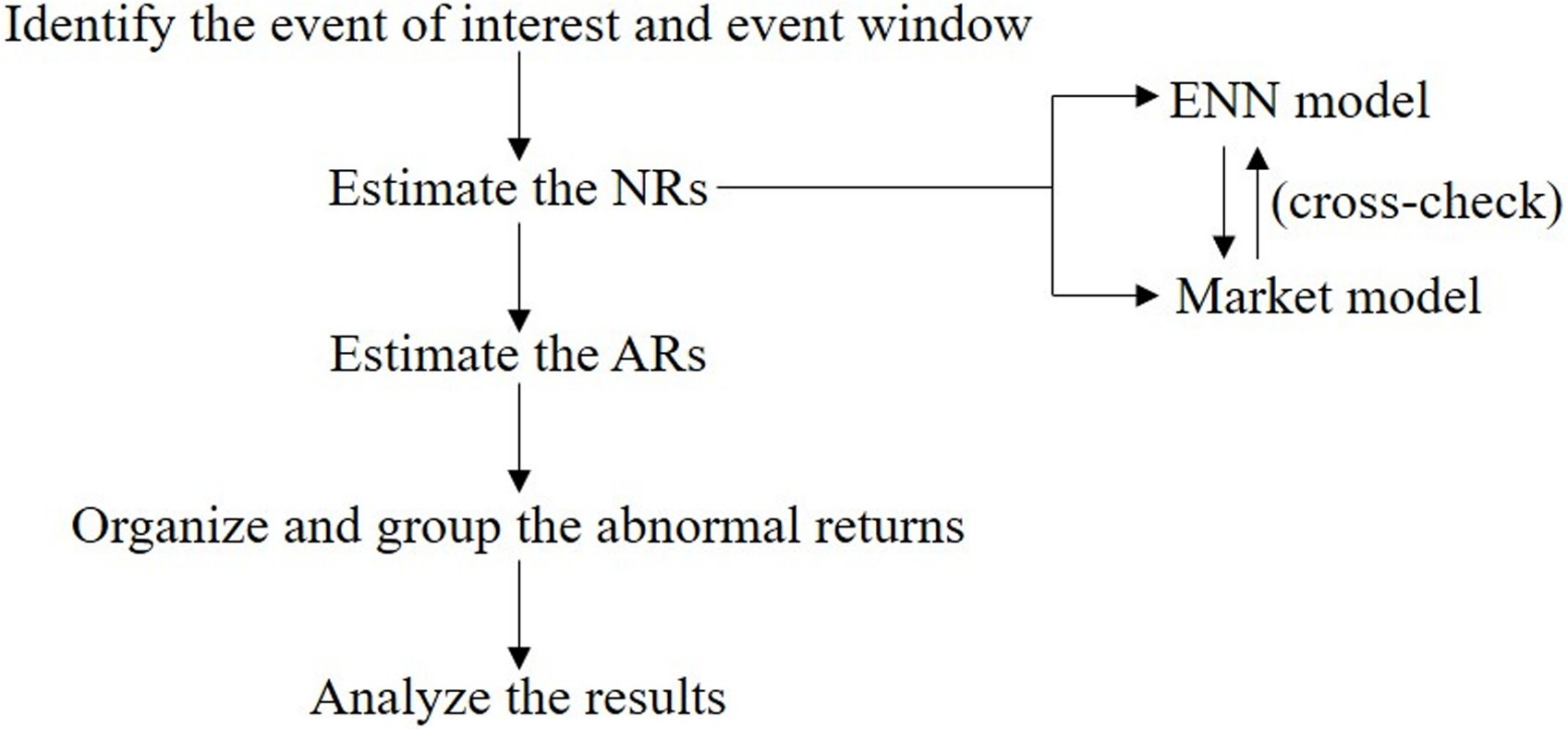
The steps of the event study method in this article.

As shown in Fig 3, the ENN model is applied to predict the NR and then calculate the AR. The market model is used to cross-check the measurement results of the ENN model. The predictive performance of these two models is assessed by two factors: the determinable coefficient (*R*^*2*^) and the root mean squared error (*RMSE*). *R*^*2*^ is used to measure the goodness of fitting. *RMSE* is used to measure the deviation between the predicted values and the realized (true) value. The prediction performance is better when the value of *R*^*2*^ is bigger and *RMSE* is smaller. The model design of ENN and the market model adopts Matlab2017a and Stata15, separately.

We take the first announcement day of the criminal detention event as the event day (day 0). If a listed company issues an after-hours announcement, the day 0 price should be shifted to the next market day. In case that no shifting is performed, abnormal returns could be biased [58, 59]. In addition, [−3,3] around the event day are taken as the event window, and 150 trading days before the event window are taken as the estimation window.

### Market model


$$R_{it}=\alpha_{i}+\beta_{i}R_{mt}+\varepsilon$$ (1)

$$NR_{it}={\hat{\alpha }}_{i}+{\hat{\beta }}_{i}R_{mt}$$ (2)

As shown in Eqs (1) and (2), *R*_*it*_ and *R*_*mt*_ refer to the individual stock return and market return on the trading day *t*, respectively. Where, the rate of return is calculated according to the formula: $R_{t}=\frac{p_{t-}p_{t-1}}{p_{t-1}}$, *P* is the daily stock price. *α*_*i*_ is a constant, *β*_*i*_ is the systematic risk parameter which is equal to the slope coefficient in a time series regression of *R*_*it*_ on *R*_*mt*_. In this paper, the CSI300 index is taken as the market index.

After obtaining the estimates of the above two parameters, the abnormal return of an individual stock can be calculated following Eq (3).


$$AR_{it}=R_{it}-NR_{it}$$ (3)

Subsequently, the cumulative abnormal return of an individual stock within the event window [*τ*_1_, *τ*_2_] can be calculated by Eq (4).


$$CAR_{\left[\tau_{1},\tau_{2}\right]}=\sum_{t=\tau_{1}}^{\tau_{2}}AR_{it}$$ (4)

Finally, the average excess return and the average cumulative excess return for the entire sample firms are obtained according to Eq (5) and formula Eq (6).


$$AAR_{t}=\frac{1}{N}\sum_{i=1}^{N}AR_{it}$$ (5)

$$ACAR_{t}=\frac{1}{N}\sum_{i=1}^{N}CAR_{it}$$ (6)

#### ENN model

When constructing an ENN model, selecting the appropriate input data to train the model is crucial. Previous research has found that the correlation between individual stock returns and market returns was stronger than that between individual stock returns and their historical returns [52]. The sample firms’ daily stock returns and market returns are selected as experimental datasets. The total number of data points in each dataset is 157. The dataset is divided into a training set and a testing set, i.e., the first 150 days before the event window for training and the rest 7 days for testing. Stock normal returns are predicted using past market returns.

In order to get a more accurate NR and provide a more credible benchmark for calculating market reaction, extensive experiments are carried out. Trainlm is selected as the training function. It refers to adjusting the weight values using Levenberg-Marquardt optimization algorithm and is one of the fastest training algorithms for medium-sized networks. The maximum training iterations is 3000 and the training error threshold is 0.00001. The iteration process will stop when the Mean Square Error *(MSE)* reaches the error tolerance. If the error tolerance is not reached after 3,000 iterations, then the parameters, such as the weight and activation function corresponding to the minimum *MSE*, are taken as the optimal solution. In this paper, we use the same training function and parameters, but there is one ENN for each stock.

## Results

### Predictive performance of the NR estimation models

In our sample, 29 trainings stop after 3000 iterations, and 2 trainings stop after the threshold reached. The predictive performance of the Elman model and the market model is reported in Table 4. The average *R*^*2*^ of the Elman model is 0.9857 and the average *RMSE* is 0.0035, while the average *R*^*2*^ of the market model is 0.2090 and the average *RMSE* is 0.0209. It demonstrates that the explanation and prediction ability of the ENN model is stronger than the market model.

**Table 4:** Average testing *R*^*2*^ and *RMSE* for the two models.

Event window	ENN model	Market model
*R* ^*2*^	0.9857	0.2090
*RMSE*	0.0035	0.0209

**Fig 4:**
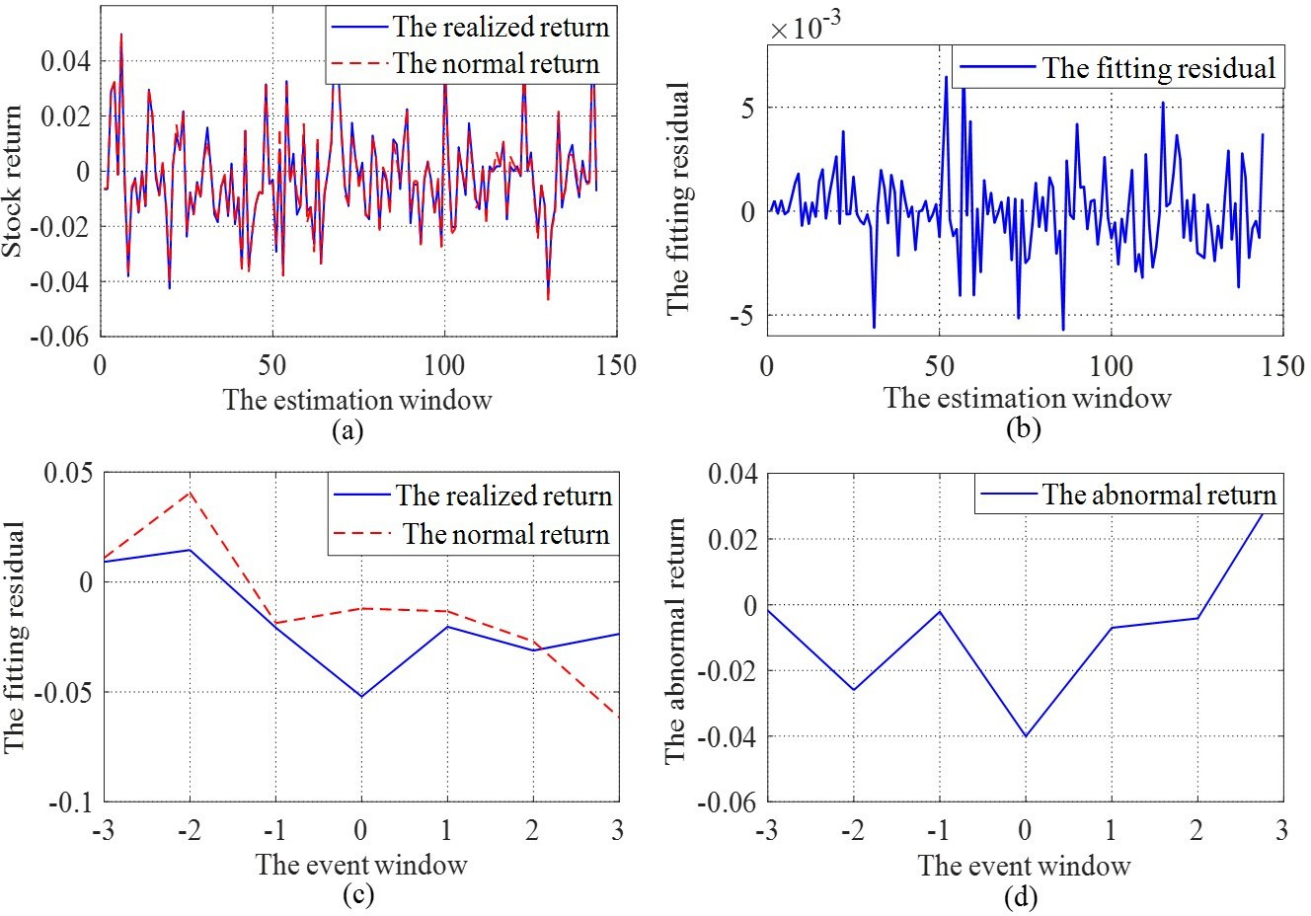
Exercising and estimating results of the ENN model. This figure plots the calculating process of the ENN model for four sectors: (a) The realized return and the normal return estimated by the ENN model in the estimation window of an individual firm; (b) The fitting residual in the estimation window; (c) The realized return and the normal return estimated by the ENN model in the event window; (d) The abnormal returns of an individual firm.

Fig 4A and 4B give the fitting effect and residual error in the estimation window with a single company as an example, respectively. Fig 4C shows the NRs of an individual company in the event window predicted by the ENN model. The difference between the NR and the realized return is the company’s abnormal return caused by the controlling person’s criminal detention announcement (Fig 4D).

### Measurement results of the market reaction

This paper measures the stock price reaction in the [−3,3] window around the criminal detention announcements of listed companies’ controlling persons using the ENN and market models. The results are shown in Table 5.

**Table 5:** ARs caused by the criminal detention announcement of controlling persons.

Event window	ENN model		Market model	
	Mean	SD	Mean	SD
-3	-1.37%	0.1214	-0.53%	0.0296
-2	-1.23%	0.0935	-0.29%	0.0358
-1	-0.70%	0.1176	-0.63%	0.0437
0	-6.67%***	0.0853	-5.52%***	0.0515
1	0.85%	0.1184	-2.15%**	0.0558
2	0.92%	0.1011	-1.50%*	0.0462
3	3.51%	0.2006	-1.92%***	0.0371
Obs	31		31	

Note: ***, ** and * are significant at the level of 1%, 5% and 10% respectively (two-tailed).

The 2–3 columns of Table 5 present the measurement results of the ENN model. The mean ARs on the 3 days before day 0 are insignificant, which indicates the criminal detention news isn’t leaked out before the first disclosure. Whereas the mean AR on day 0 is -6.67% and significant at 1% level (p = 0.0001), indicating the stock prices have a significant negative reaction to the criminal detention announcements of controlling persons on the event day. For the remaining days after day 0, the mean ARs of the sample companies are not statistically significant, which means that the market reaction to the criminal detention announcements of controlling persons has almost disappeared.

The 4–5 columns of Table 5 report the measurement results of the market model. Similar to the results of the ENN model, the market reaction on the event day is also significantly negative, but the reaction level is lower to -5.52% (p = 0.0001). Different from the results of the ENN model, the mean AR after the event day is also significantly negative. However, with the development of information technology and the continuous improvement of investor quality, investors are increasingly quick to react to new information, and the reaction time is gradually shortened. Therefore, this reaction may be caused by other new information emerging after the event day and can no longer be regarded as the market reaction to the criminal detention announcements of controlling persons. From this, we can judge that the reaction time of the Chinese stock market to controlling persons’ criminal detention announcements is only 1 day.

### Robustness test

In applying the event study method, it is crucial to control the influence of confounding events. Some other major events might have occurred on the announcement day of the criminal detention. In addition, the firms will carefully consider the time and manner of disclosing information about crisis events and may issue other news to distract investors’ attention to mitigate the negative impact on their stock prices. In order to ensure that the observed stock price changes are all caused by the disclosure of the criminal detention events of controlling persons and not by other material information, we complete confounding event checks.

The checking process follows the procedure identified by Hammersley et al. [60]. We read all announcements issued by the sample firms on the event day, classify their content, and control for confounding events. As reported in Panel C of Table 3, we find that 19 companies do not issue any other announcements, or the content of other announcements issued do not constitute confounding events, such as chain events announcements and procedural announcements. The former includes the share frozen of the controlling person and the resignation of the chairman of the board of directors held by the controlling person. The latter includes the stock suspension/resumption notice, the conference notice, and the independent directors’ debriefing report. The information in the above announcements has been included in the investors’ expectations about the future risk level and the firms’ profitability and does not provide new valuable information. In our sample, the announcement content that constitutes confounding events mainly includes massive disclosure of other information to confuse the public on the event day, simultaneous disclosure of criminal detention information and other significant good news, and timely disclosure of the response measures after the criminal detention of controlling person. Based on these analyses, we use the 19 companies mentioned above without confounding events as a sample to test the robustness of our results. The results of the robustness test are displayed in Table 6.

**Table 6:** ARs of the firms without other material information.

Event window	ENN model		Market model	
	Mean	SD	Mean	SD
-3	2.50%	0.1028	-0.49%	0.0332
-2	-2.48%	0.1094	-0.45%	0.0428
-1	-0.96%	0.1121	-0.30%	0.0484
0	-8.04%***	0.0772	-5.82%***	0.0490
1	0.33%	0.0774	-0.85%	0.0587
2	1.68%	0.1081	-1.39%	0.0445
3	4.27%	0.2426	-2.00%***	0.0292
Obs	19		19	

Note: *** is significant at the level of 1% (two-tailed).

The market reaction measured by the ENN models is still significantly negative on the announcement day, but the reaction level is higher than the results of 31 companies of -6.67% to -8.04%. In addition, the result measured by the market model is still lower. Table 4 also shows that the significance of the measurement results of the ENN model is consistent with Section 4.1. However, in the measurement results of the market model, the ARs on the first and second day after day 0 are no longer statistically significant, which again shows the reaction time of the Chinese stock market to controlling persons’ criminal detention announcements is only 1 day. The above results suggest that the research conclusion of this article is reliable. Moreover, it also indicates that the crisis communication measures taken by listed companies when disclosing information about the criminal detention event of controlling person are effective, but the effect is limited. Just as the saying goes, “trickery is effective, but it is limited”.

Data snooping bias is also a very common and serious problem in financial analysis. However, constructing the ENN model in this paper aims to provide a benchmark for calculating the market reaction rather than to provide a generally applicable investment strategy. This benchmark refers to the expected return if the criminal detention event had not occurred, counterfactual. Therefore, the better the predictive performance, the more accurate the calculated market reaction. What’s more, we also use the classical market model to estimate the ARs and find a negative market reaction as well, indicating that the ENN model constructed in this paper is appropriate. The methodological contribution of this paper demonstrates that the combination of ENN and event study method helps improve the measurement accuracy of the market reaction. Therefore, data snooping is not a serious issue for our research.

### Further research

The negative excess return corresponds to the wealth loss of investors caused by the criminal detention announcements of listed companies’ controlling persons. Therefore, we further analyze the changes in the circulation market capitalization of the sample firms before and after the event day.

**Fig 5:**
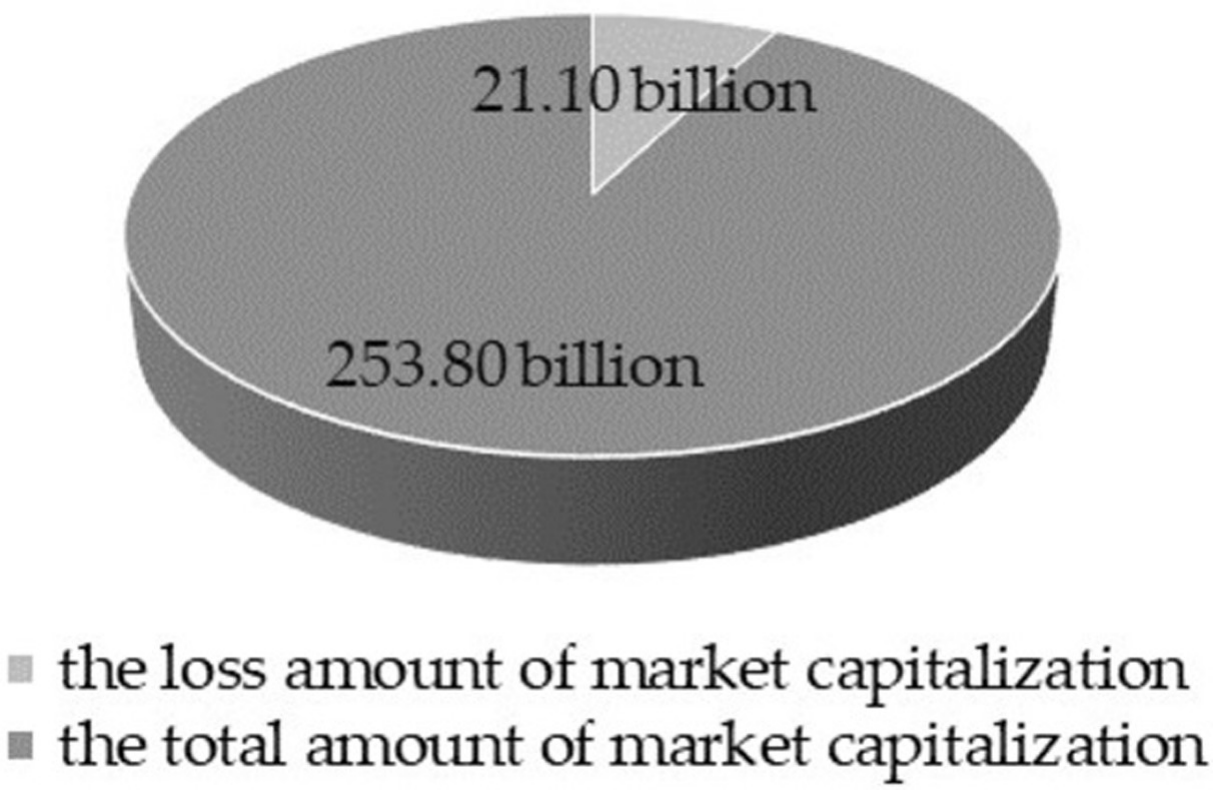
Changes in the total amount of market capitalization.

Fig 5 shows the change of circulation market capitalization of the 31 firms in our sample. The entire pie represents the total market capitalization the day before the event day (RMB 274.90 billion) when the stock price has not been affected by the criminal detention announcements. The dark sector represents the total market capitalization on the event day, which is RMB 253.80 billion. The light-colored sector represents the difference between the above two sectors, indicating the loss of market capitalization on the event day. The 31 listed companies caused a total loss of RMB 21.1 billion on the event day alone in the Chinese capital market. It is equivalent to 7.68% of the total market capitalization on the day before the event day, and the average loss of each firm reaches RMB 681.00 million. Before the merger of the Main Board and the Small and Medium Enterprise Board in Shenzhen Stock Exchange, the minimum size of the listed company in a normal trading state is RMB 1 billion. The loss of market capitalization caused by the criminal detention announcements of controlling persons is about equivalent to that of 21 small-sized listed companies disappearing from the Chinese capital market in one day. This damage cannot be underestimated.

To build a high-quality listed company, the controlling person’s high legal management consciousness and personal moral cultivation are demanded. In some sense, investors’ investment decisions are based on these two premises. The awareness of legal management of the invested company will enhance investors’ confidence and support to the company, thus cultivating a group of long-term equity investors and attracting new investors to buy their stocks. The increasing number of investors, in turn, will boost the share price and achieve the goal of maximizing shareholders’ wealth. The criminal detention of the listed company’s controlling person undoubtedly destroys this premise and foundation, which in turn causes panic among investors. They will react by selling and fleeing. This eventually leads to severe evaporation of shareholders’ wealth and a significant drop in share prices.

## Discussions and conclusions

Under the background that the frequent occurrence of the criminal detention of listed companies’ controlling persons is widely concerned by the society, this paper is the first to quantitatively analyze the effect of controlling persons’ illegalities on stock price returns in the context of Chinese listed companies. We apply the ENN model to the event study method to measure the market reaction and find a higher reaction level compared with the measurement result of the traditional market model. Concretely, the results show that the stock price has a significant negative reaction to the criminal detention announcements on the event day, and the average negative reaction level is -6.67%. Furthermore, when considering only the sample of 19 firms without other material information, we uncover the average AR on the announcement day is more negative with an increased reaction level of -8.04%. It indicates that listed companies’ crisis communication measures can mitigate the negative impact of such mandatory disclosure information on their stock prices, but the effect is limited. Moreover, the 31 companies in our sample cause a total loss of RMB21.1 billion in market capitalization on the announcement day alone. According to the minimum size of companies listed on the Shenzhen Stock Exchange at the end of 2020, the loss is approximately equivalent to the disappearance of 21 small listed companies from the Chinese capital market.

Overall, our study reveals that the impact of controlling person’s illegalities of listed companies on the sustainable development of listed companies and the smooth operation of the stock market is significant, both on the statistical and economic levels, although the group of controlling persons is small. Being detained seriously damages the legitimate rights and interests of investors.

The controlling person is always the core factor in the operation, development and governance of a listed company, determining the trajectory of the listed company’s behavior and style of acting. Therefore, improving the controlling person’s legal consciousness and the moral level is indispensable and important for the sustainability of a listed company. First, as the ultimate decision-maker of a listed company, controlling person’s behaviors should be supervised by the public and further improve information transparency. Second, the punishment of the controlling person should be strengthened, and they should bear unlimited compensation liability for the losses of other investors. Finally, legislation should be strengthened to enhance legal deterrence and prohibit the controlling person with serious illegal records from serving as the controlling person of other listed companies. Only by using high costs can we build a “high-tension line”. This is a necessary measure to reduce the controlling person’s illegalities.

China is the largest emerging market and the second-largest economy in the world. The findings of this paper may guide decision-making for the subsequent regulations and have important implications for other countries. Moreover, our study focuses on exploring the applying of ENN into the event study method and demonstrates that the combination of them helps to improve the measurement accuracy of the market reaction. It provides guidance for most researchers to further explore the application of data mining intelligence method in nonlinear problems.

Based on the results discussed above, we would like to give some outlooks to driving future research opportunities. First, due to the limitation of sample size, more in-depth analysis about this topic requires the accumulation of more data over time, such as the difference of reaction levels caused by different crime types and the influencing factors of the severity of the negative reaction. Second, a standard ENN model effectively forecasts the stock return based on the nonlinear mapping. More research could be performed to further optimize the forecast accuracy of the normal return. Like using other matured recurrent neural network models or exploring whether DIOCs is needed in the mapping relationship between the individual stock returns and the market return series.

## Supporting information

S1 Table The criminal detention announcements of listed companies’ controlling persons. (XLSX)

S2 Table Stock price data for calculating the market reaction. (XLSX)
